# Nutritional status and dietary intake among Nigerian adolescent: a systematic review

**DOI:** 10.1186/s12889-024-19219-w

**Published:** 2024-07-02

**Authors:** Hadiza Abdullahi Abubakar, Mohd Razif Shahril, Sumaiyah Mat

**Affiliations:** 1 Center for Healthy Aging and Wellness, Faculty of Health Sciences, Universiti Kebangsaan Malaysia, Kuala Lumpur, Malaysia; 2 Faculty of Basic Medical Sciences, Bayero University, Kano, Nigeria

**Keywords:** Adolescent, Undernutrition, Overnutrition, Dietary intake, Nigeria

## Abstract

**Introduction:**

The prevailing nutritional conditions and the triple challenge of malnutrition faced by adolescents have adverse consequences for both the present and future generations’ health and nutrition. Summarizing the available research on the nutritional status and dietary habits of adolescents in Nigeria is crucial.

**Objective:**

This study aims to systematically evaluate available literature on the nutritional status of adolescent aged 10 to 19years in Nigeria.

**Methodology:**

A systematic search using PRISMA guideline was conducted. Three electronic databases were searched i.e., PubMed, Web of Science and Scopus using specific terms and keywords for online articles published between 2013 and 2023. After applying specified inclusion and exclusion criteria, 51 articles were selected for data extraction, synthesis and quality assessment.

**Results:**

Of the 51 included studies, 78.4% were conducted in the Southern Nigeria, 11.8% in the Northern Nigeria and 9.8% included both regions. The prevalence of overweight ranged between 0.8 and 31% and obesity ranged between 0.1 and 14%. The prevalence of thinness, stunting and underweight ranged between 3 and 31%, 0.4 to 41.6%, 0.3 to 73.3% respectively. The review also identified an inadequate intake of essential nutrients including iron, zinc, calcium, vitamin A, C, D, niacin, thiamin, riboflavin, cobalamin, and folate, with vitamin A deficiency prevalence ranges from 44 to 96%. The dietary patterns were characterized by a high consumption of cereals grains and starchy foods, low animal proteins, fast-food with soft drinks, and limited consumption of fruits and vegetables along with meal skipping.

**Conclusion:**

These findings portray a complex picture of the nutritional challenges faced by this demographic group, highlighting both undernutrition and overnutrition, poor eating behaviour and micronutrient deficiency as significant concerns. The review revealed regional disparities in research representation, with a concentration of studies in Southern Nigeria. This highlights the importance of directing research efforts toward the northern regions, where the prevalence of nutritional issues is equally severe, but less studied.

**Systematic review registration number:**

PROSPERO CRD42023481095.

**Supplementary Information:**

The online version contains supplementary material available at 10.1186/s12889-024-19219-w.

## Introduction

Adolescence is a critical period of physical, psychological, and social development, and is characterized by rapid growth and increased nutritional demands [1]. This stage is sensitive to malnutrition owing to the increased physiological need for nutrients [2]. Adequate nutrition during this phase is essential for achieving optimal physical and cognitive development, as well as for laying the foundation for healthy adulthood [1]. However, adolescents worldwide face numerous challenges related to nutrition, leading to a wide range of nutritional issues with long-term implications for their health and wellbeing [3–6]. Globally, adolescents bear a significant burden of malnutrition particularly in low- and middle-income countries. According to recent data, malnutrition affects approximately 8% of adolescent girls (49 million) and 10% of women (154 million) globally [7]. In Africa, the burden of malnutrition among adolescents is particularly pronounced, with a substantial proportion of adolescents experiencing both undernutrition and overnutrition. [3, 4, 8]. In Nigeria, as in many other low- and middle-income countries, adolescents constitute a significant proportion of the population [9], and their nutritional status and dietary intake are crucial determinants of the nation’s future health and productivity. Nigeria is no exception to global malnutrition, and nutrition-related diseases remain a major public health concern. Although malnutrition is a countrywide issue, its prevalence varies by geopolitical zone, and higher-risk populations tend to be in the poorest and most remote regions [10]. With a diverse population, varying socioeconomic conditions, and cultural differences, Nigeria presents a complex landscape for understanding the nutritional status and dietary intake of its adolescent population.

In many African countries, rapid nutrition transition has led to an emerging triple burden of malnutrition, where undernutrition and micronutrient deficiencies persist alongside the rising burdens of overweight and obesity [11]. Food insecurity, poverty, and limited access to health services contribute to these issues [3]. Multiple forms of malnutrition are threatening the health, development and learning of school-age children and adolescents in Africa. About 7% of school-age children and adolescents are underweight, while more than 13% are living with overweight [12]. All forms of malnutrition in women and girls, including underweight, anaemia and overweight have serious consequences for women’s health and well-being [12].

According to the Nigerian Demographic and Health Survey (NDHS) in Nigeria [10], undernutrition and micronutrient deficiencies are more prevalent in the northern part of the country than in the south. 12% of women aged 15-19years are thin (BMI-for-age), and 28% are overweight or obese. More than half (58%) of the women had some degree of anemia. 28% each are mildly and moderately anaemic, and 2% are severely anaemic [10]. Both over- and undernutrition across the adolescent years have implications on physical and cognitive development, with associated risks for school attendance, academic performance, and productivity in adulthood, as well as for susceptibility to developing non-communicable diseases in later life [11, 13]. Adolescents who are malnourished are more likely to experience obstructed labor and other obstetric complications as well as give birth to children with intrauterine growth restriction, low birth weight, preterm delivery, higher infant mortality, and poor child outcomes [14]. Children of malnourished women are more likely to have cognitive impairments, short stature, lower resistance to infections, and a higher risk of disease and death throughout their lives [15]. Therefore, if an adolescent girl enters the reproductive cycle in a malnourished state, she will grow up into a malnourished adult and give birth to a malnourished child, contributing to an unproductive community and the cycle of intergenerational transfer of malnutrition [16]. Improving nutrition among adolescents is critical in the improvement of the nutritional status of the entire population. While Nigeria has made substantial efforts to improve child nutrition over the years, there is a growing concern that the nutritional needs of adolescents have not received adequate attention [17]. This systematic review aimed to provide a comprehensive overview of the nutritional status and dietary intake among adolescents in Nigeria by synthesizing findings from a wide range of studies. This will provide a foundation for future research aimed at improving the nutritional status and well-being of Nigerian adolescents, thereby promoting a healthier and prosperous future for the nation.

## Method

### Eligibility criteria

The study protocol was registered in the International Prospective Register of Systematic Reviews (PROSPERO) with registration number CRD42023481095, in accordance with Preferred Reporting Item for Systematic review and Meta-analysis (PRISMA) guidelines [18]. The Population, Intervention, Context, and Outcome (PICO) framework was applied to ensure a systematic approach in capturing relevant articles. The study population focuses on adolescents aged 10 to 19 years in the context of Nigeria, without any specific interventions. The age of 10 to 19 years is a period in human growth and development that occurs after childhood and before adulthood, characterized by increased nutritional demands, making it pivotal stage establishing lifelong health habit. Adequate nutrition during this phase is essential for optimal physical and cognitive development, lying the foundation for healthy adulthood [1]. The primary outcomes of interest encompass the prevalence of stunting, thinness, underweight, overweight, and obesity, as well as various aspects of dietary behavior, including dietary patterns, eating habits, dietary diversity, food consumption, and nutrient intake.

The selection of articles was made based on pre-determined inclusion and exclusion criteria. The inclusion criteria set were: (1)] Adolescents 10 to 19years (2) Types of study: Cross-sectional or prevalence (3) Outcome measures; nutritional status such as thinness, stunting, wasting, underweight, overweight, obesity, dietary intake, eating behaviour, dietary diversity, nutrient deficiency (4) Written in English and published from 2013 to 2023. Studies involving adolescents with diseases, intervention and review articles were excluded from this review.

### Search strategy

The literature search was conducted in April 2023. Keywords and search terms were identified from the title and main objectives of the study. A comprehensive search string was developed based on the review’s objectives. The search string, composed of keywords and their synonyms, was constructed using Boolean Operators to search each respective database. The search string is as follows:

((adolesc* OR teenager* OR teen* OR youngster* OR youth) AND (“nutritional status” OR malnutrition OR undernutrition OR stunting OR thinness OR thin OR wasting OR “micronutrient deficiency” OR “mineral deficiency” OR “vitamin deficiency” OR overweight OR obese* OR “high body mass index” OR “high BMI-for-age” OR “high BMI” OR overnutrition OR diet OR “dietary pattern” OR “dietary practice” OR “healthy eating” OR “eating habit” OR “food recall” OR “diet recall”) AND Nigeria) NOT pregnan*.

Three electronic databases were searched which were PubMed, Scopus, and Web of Science. Additionally, grey literature was explored through Google Scholar. All identified articles were imported into Mendeley reference manager application for further management. Subsequently, all the articles were combined into one single shared folder for review.

### Selection of studies

The initial search strategy was obtained through discussion between the authors (H.A.A, S. M and M.R.S). Firstly, all duplicates were removed, and the remaining articles were screened for eligibility based on their title and abstract. This was carried out using an online systematic article management software, Rayyan Ai, to facilitate our screening process [19]. Full texts of the remaining results were then screened for eligibility according to the inclusion and exclusion criteria. Any potentially eligible article was read in full. Relevant studies were selected, and data were extracted into a standardize form for quality assessment and evidence synthesis.

The use of two reviewers at the study selection stage reduced the chance of missing out important information and allowed the assessment to be done independently with different points of view. Any discrepancy found in the process was resolved through consensus and contacting the authors of the original study, if deemed necessary. Duplicate assessment of the studies would decrease the possibility of rejecting relevant studies and would give the greatest benefit in selecting or rejecting studies that required difficult judgments [20].

### Data extraction

The key variables extracted from all the relevant research articles that met the inclusion/exclusion criteria in this review were : author and year of publication, state that the research took place in (study location), sample size, age range of participants, sex, study setting, specific nutritional indicators such as prevalence of thinness, stunting, underweight, overweight and obesity. Additionally, we extracted information on dietary intake, eating behaviours and nutrients deficiencies where available.

### Data synthesis

The extracted data were then synthesized for analysis, and we presented the result narratively. Available data from all studies were considered with no regard to the methodological quality of each study. For anthropometric indices, the results were assessed according to each specific parameter. Prevalence rates are presented in ranges to provide a visual understanding of the distribution of nutritional status. The synthesis described the dietary behaviours and pattern narratively.

### Quality assessment

The quality of the included study was assessed using the National Institutes of Health (NIH) checklist for observational and cross-sectional studies, due to its comprehensive criteria that are specifically designed to evaluate the methodological rigor of observational and cross-sectional studies [21]. This tool provides a standardized approach to assess the quality and to ensure consistency in the evaluations process across multiple studies. It was designed to assist in focusing on the essential aspects for evaluating the internal validity of cohort and cross-sectional studies. Consequently, the tool could appraise the ability of each study to draw associative conclusions regarding the impact of the examine exposures on outcomes. It comprises 14 criteria that can be assigned scores such as “Yes”, “No”, “Cannot Determine”, “Not Applicable”, critical for assessing study quality, including the clarity of research question, selection and definition of study population, validity and reliability of measurement, and consideration of confounders [21]. Each criterion carries equal weight in determining the overall grade for each study, presented as the total score. After answering a series of 14 questions, the quality of each study was reported as poor, fair, or good. Quality was rated as ‘0’ for poor (0–4 out of 14 questions), ‘I’ for fair (5–10 out of 14 questions), or ‘II’ for good (11–14 out of 14 questions) [22].

### Inter-rater reliability of the quality assessment process

The quality assessment process was conducted by two authors (H.A.A and S.M). To ensure the reliability of our quality assessment, we calculated the inter-rater reliability using Cohen’s Kappa coefficient. The Kappa coefficient for our quality assessment was 0.70, indicating substantial agreement between the raters. During the process, any discrepancies in scoring were resolved through consensus discussions. This ensured that all assessments were consistent and accurate, enhancing the reliability of our quality evaluation.

## Results

### Qualitative synthesis of the studies

**Fig. 1:**
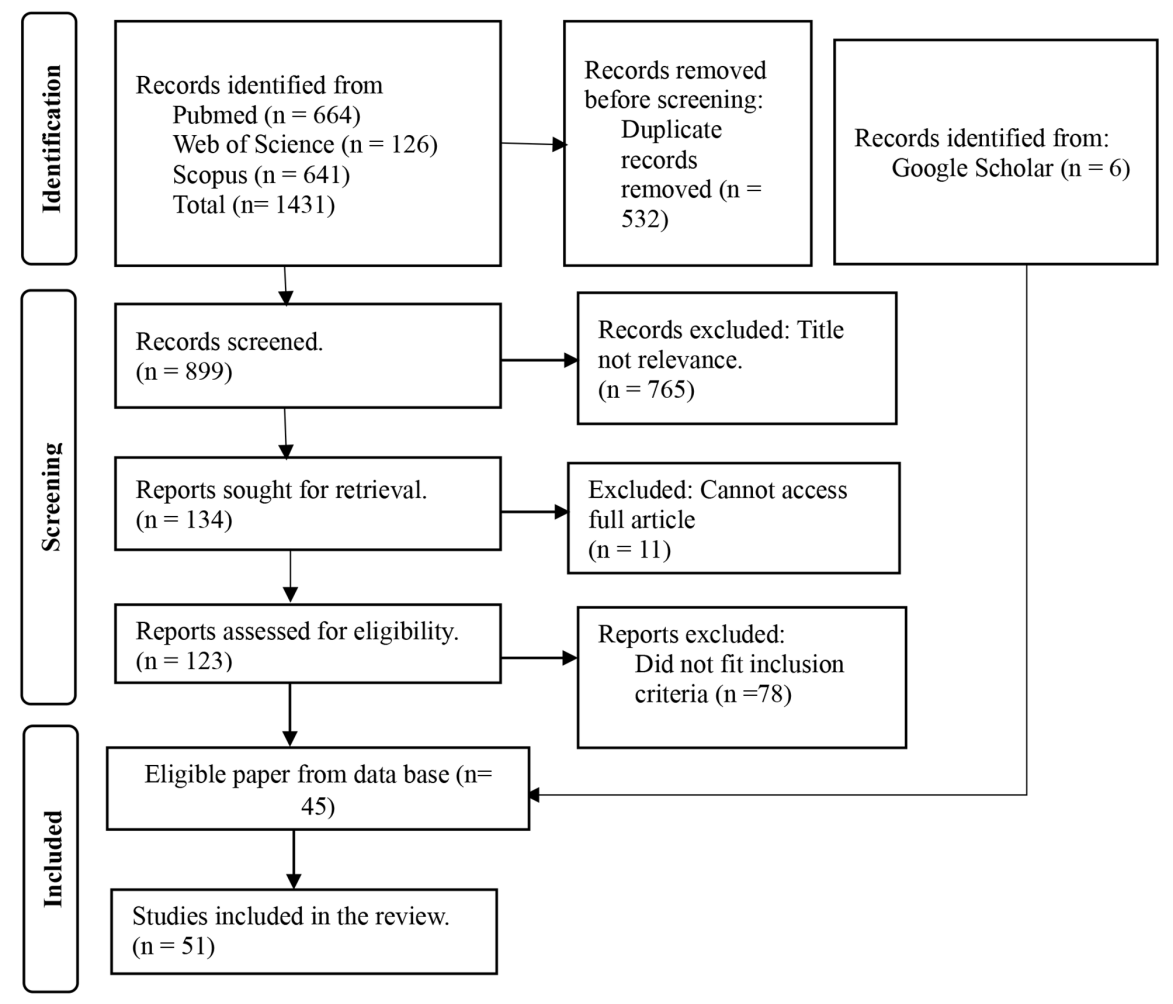
Flow diagram of the study selection process

A total of 1431 articles were identified from the selected database and six from google scholar. After the removal of duplicates, 899 articles were screened by title and abstract. Consequently, 765 articles were excluded, leaving 134 for full-text assessment. After reading the full texts, 89 studies were excluded with reasons: cannot access full article (*n* = 11) and 78 articles did fit inclusion criteria. Finally, a total of 51 studies were included for qualitative synthesis. Figure 1 presents a flow diagram of the study selection process. The data extracted from the 51 articles included in this review are summarized in Additional file 1.

### Quality of studies

The methodological quality assessment of each study using the NIH Quality Assessment Tool presented a score variation between six and nine points. Considering all studies that have been included in this systematic review, eight studies achieved a score of nine, 16 studies achieved a score of eight, 25 studies achieved a score of seven and two studies achieved a score of six (Additional file 2). The quality of studies included in this systematic review was fair, which was mostly explained by the observational study design. Some of the questions in the NIH quality assessment tools were ‘Not Applicable’ to most of the studies include such as ‘Loss to follow-up’, ‘Measurement of confounding variable’ and ‘outcome assessors blind to the exposure’ (Additional file 2). Some studies also did not report sample size justification. However, this was mostly due to the exploratory nature of observational studies and was not considered a fatal flaw [21]. Additional file 2 shows the NIH quality assessment scores for the included study.

### Nutritional status

**Fig. 2:**
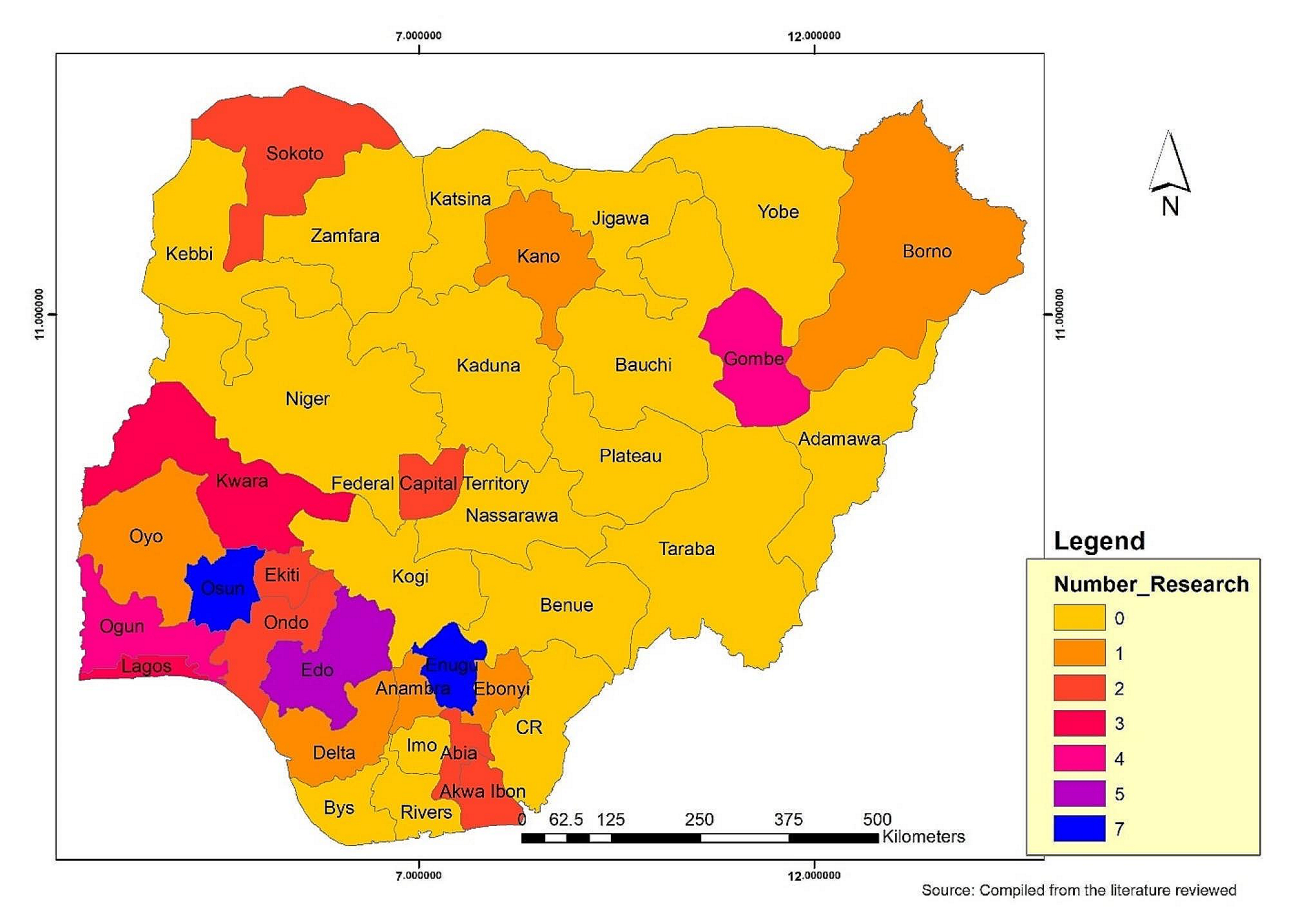
Distribution of study according to state

Of the 51 studies included, 50 employed a cross-sectional design, with only one being a qualitative study [23]. The earliest studies were published in 2013 (24–28), while the most recent ones were published in 2022 [13, 23–25]. Forty-two studies were conducted in a school setting [11, 23, 26–63], while nine were community-based [13, 24, 64–70]. Geographically, 40 (78.4%) studies were conducted in Southern Nigeria, while only six (11.8%) studies [31, 33–35, 64] were conducted in Northern Nigeria, and five [13, 24, 36–38] (9.8%) were combination of both. The sample size in each cross-sectional study ranged from 90 [39] to 9242 [37]. Out of the 51 studies, 46 included both male and female participants, while five studies specifically involved only females [25, 28, 31, 37, 40]. Ten articles reported prevalence of thinness [11, 24, 36, 38, 41–46], six reported wasting [30, 33, 35, 47–49], 13 reported stunting [30, 32, 33, 35, 36, 41, 42, 45–50], 21 reported underweight [13, 26, 29, 31, 32, 36, 37, 40, 42, 46, 47, 50–59], 29 reported overweight [26, 27, 29, 31–36, 43–47, 49, 50, 52–64], and obesity was documented in 26 studies [26, 27, 29, 31–36, 43, 45–47, 52–66]. In contrast, five studies [25, 39, 41, 42, 45] reported nutrient intake, and dietary patterns were examined in 16 studies [23, 25–29, 32, 35, 39, 40, 50, 55, 65–69]. The majority of included studies included originated from Enugu state (*n* = 7), followed by Edo state (*n* = 5), and Ogun and Oyo state, with four studies each (Fig. 2.). Figure 2 shows the distribution of study according to state.

**Fig. 3:**
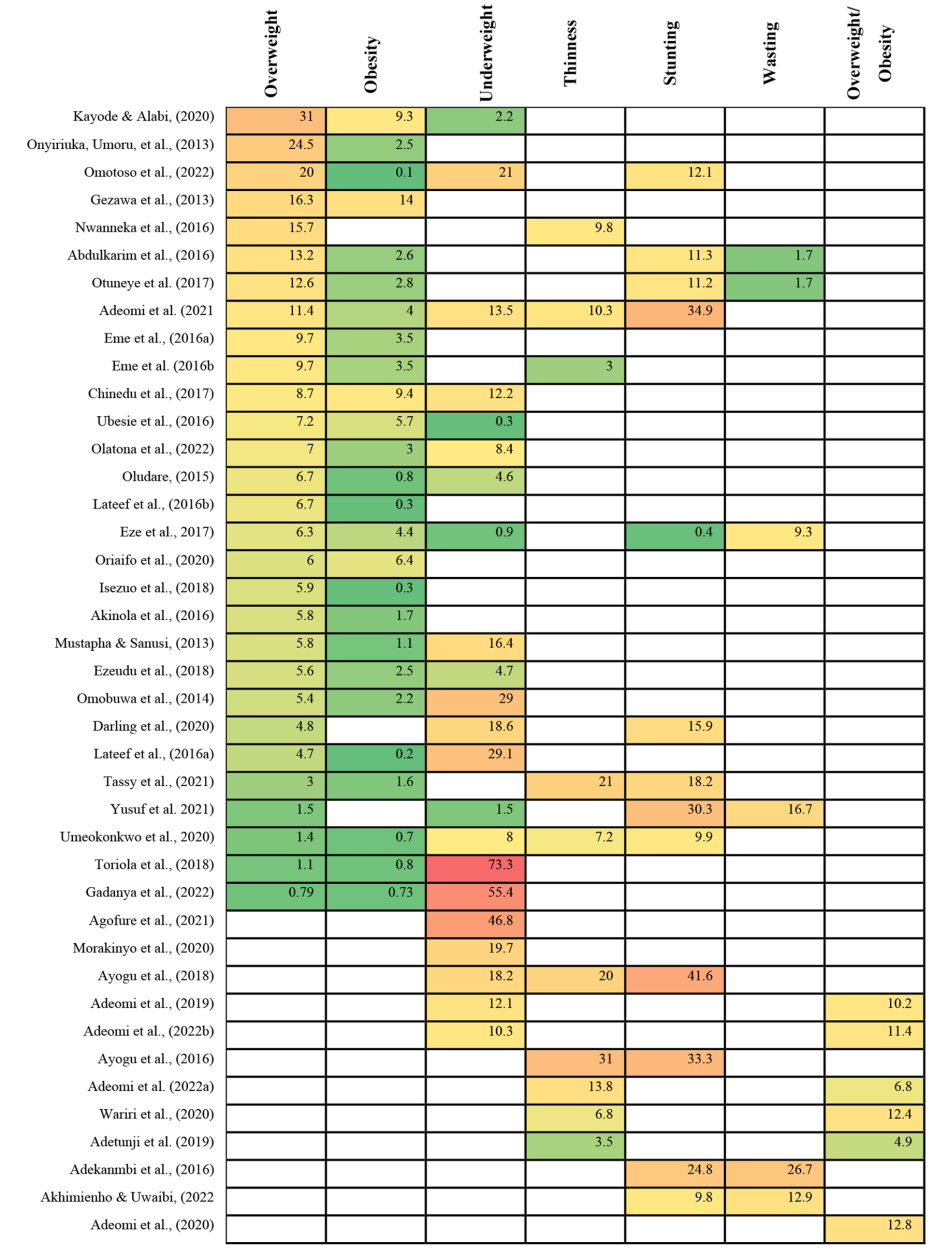
Prevalence of nutritional status of the included studies. **The colour gradient**: Green indicates lower prevalence, Red indicates higher prevalence, and yellow falls in the middle

The reported prevalence of thinness varied between 3 and 31%, and for wasting, it ranged from 1.7 to 26.7% (Fig. 3). Notably, all the 10 studies were conducted in the southern states with only three included data from northern states [24, 33, 49]. Among the combine studies, thinness was more prevalent in the northern states compared to the southern states. Prevalence of thinness was higher among males (8.7%), individuals attending public schools (8.6), and those residing in rural parts of the metropolis (14.3%) [46]. Moreover, stunting and wasting was more prevalent among males than females, and those who skipped meal and those who took little fruits and vegetable [47]. The prevalence of stunting was reported in 13 studies, ranging from 0.4 to 41% (Fig. 3). Among these studies, nine were conducted in the southern region [30, 32, 41, 42, 45–48, 50], three in the northern region [33, 35, 49] and one included data from both regions [66]. Notably, in the study that included both regions, the prevalence of stunting was higher in the north (47.2%) than in the south (21%). Underweight prevalence ranged from 0.3 to 73.3% (Fig. 3). Among the 21 studies that reported underweight, one was conducted in northern states (Kano state) [31], while 20 were conducted in the southern states [13, 26, 29, 31,, 36, 37, 40, 42, 46, 47, 50–59], with only three included data from northern states [13, 36, 37].

A total of 29 studies reported the prevalence of overweight, which ranged between 0.8% and 31% (Fig. 3). The highest overweight prevalence was reported in the Southern state such as Osun [54], Edo [27], and Kwara [32], while lowest value was reported in Northern state such as Kano [31] and Sokoto [49]. The obesity prevalence ranged from 0.1 to 14%. Most of the northern state studies reported low obesity prevalence such as Kano [31], Sokoto [34], and Abuja [33], while southern state reported higher prevalence such as Ogun [52], Osun [54], and Edo [63]. Conversely, Borno state is in the Northern Nigeria but reported the highest prevalence of obesity of 14% [64]. Figure 3 shows the prevalence of nutritional status of the included study.

### Nutrients intake

All the five studies that reported nutrients intakes were conducted in Southern Nigeria. No study was conducted in Northern Nigeria. Low levels of protein, iron, zinc, calcium, riboflavin, thiamine, niacin, vitamin A, vitamin C, and vitamin D were observed. While the majority of the participants had sufficient carbohydrate intake, neither males nor females met the Recommended Nutrient Intake (RNI) for energy and protein. Among these five studies, three studies [41, 42, 45] reported prevalence of vitamin A deficiency among adolescents ranged from 44 to 96%. Regarding iron intake, one study [41] identified 64% of participants as anaemic, another [25] reported 43.3% of adolescent female being iron deficient and Tassy et al., [45] found that 91% had intake below estimated average requirement (EAR). Adolescent girls exhibited low dietary iron intake, with non-heme iron surpassing heme iron among those with iron deficiency. For zinc deficiency, one study [42] reported a rate of 43.3% among the adolescents, while another [45] found intake below EAR in 58% participants. Other two studies [39, 45] highlighted inadequate energy intake (1590Kcal/day and 1758.5 ± 472.2 for 10 to 12years and 1833.3 ± 627.7 for 13 to 15years respectively) and low nutrient intakes. Specifically, 85.6% and 28% had insufficient intake for niacin, 76.7% and 99% for calcium, 72.2% and 74% for riboflavin, 54.4% and 7% for protein, 32.2% and 75% for thiamine, and 15.6% and 79% for vitamin C respectively. Moreover, 99% had low intake for Vitamin D, 58% for vitamin B12 and 99% for folate [45]. These findings collectively indicate that nearly everyone in the studied population had an intake below the expected average requirement (EAR).

### Dietary pattern

Sixteen studies reported dietary intake, patterns, and attitudes of adolescents. The prevalent dietary pattern was characterized by a high consumption of cereals and starchy foods, including yam, cassava, sweet potatoes, rice, maize, breads, and legumes. Conversely, there was a lower intake of meat, fish, milk, poultry, seafoods, organs meat and egg. The review highlighted that the adolescent typically consumed three meals daily. Dinner emerged as the most frequently consumed meal, followed by lunch and breakfast. Meal skipping was a common occurrence, attributed to time constraints, lack of appetite and not feeling hungry. Among all meals, breakfast was the most frequently skipped, and it was more common among girls than boys. Furthermore, snacking and soft drink consumption were widespread. Regular consumption of snacks such as biscuits, doughnut, buns, pies and sweetened beverages, sweets, chocolates was noted with female exhibiting a higher frequency of snacking compared to males. The primary factors influencing adolescents’ food choices encompass taste, personal preferences, financial considerations, nutritional, and popularity.

## Discussion

This review summarizes the existing literature concerning the dietary intake and nutritional status of adolescents in Nigeria. The findings illuminate the multifaceted challenges faced by this demographic group. Although 51 studies were included, one notable observation from this review is the regional disparities in the distribution of the studies. Most of the research was conducted in Southern Nigeria, with limited representation from the northern regions. This disparity in research efforts reflects broader socioeconomic and academic disparities between the two regions.

The review revealed a significant prevalence of thinness (3 to 31%), wasting (1.7 to 26.7%), and stunting (0.4 to 41.6) among Nigerian adolescents. Underweight was another prominent issue identified in the review, with prevalence rates ranging from 0.3 to 73.3%. Similar finding was observed in a scoping review conducted on the nutritional status of school-age children and adolescents in eastern and southern Africa [71]. The review revealed that the prevalence of thinness, stunting, and underweight ranged as follows: 3.0 to 36.8%; 0.6 to 57.0%; 0.8 to 27.1% [71].A study among adolescent conducted in two Nigerian states revealed that a diversified dietary pattern showed an inverse relationship with thinness (OR: 0.44; *p* = 0.007; 95% CI: 0.24 to 0.80) [24]. Additionally, household size (OR 1·10; *P* = 0·001; 95% CI (1·04, ·16)) and the uppermost wealth index (rich) (OR 0·43; *P* = 0·016; 95% CI (0·22, ·86)) had a significant positive and inverse associations with thinness, respectively [13]. Factors associated with underweight include food expenditure, gender, female-headed households, and age [42, 57], while food expenditure, male gender, engaging in hawking after school, and residing rural settings were associated with stunting [33, 42]. Adolescents in public schools were more underweight than their counterparts in private schools [53]. In addition, undernutrition was higher among women who resided in the northwestern region of Nigeria and those living in rural areas [37]. These conditions can have long-lasting effects on growth and development, as well as physical and cognitive health among the adolescents [70]. While undernutrition remains a significant concern, the review also highlighted the growing prevalence of overweight (ranges from 0.8 to 31.0%) and obesity (from 0.1 to 14.0%) among Nigerian adolescents, particularly in the southern states. This is similar to the findings in East and Southern Africa, in which the prevalence of overweight and obesity ranged from 9.1 to 32.3% and 0.8 to 21.7%, respectively [71]. A doule burden of malnurition was evident in the low- and middle-income countries. This was reported in a scoping review of the nutritional status of school-age children and adolescents in seven global regions of low- and middle-income countries [6]. In their findings, stunting, thinness, anemia, and other micronutrient deficiencies persisted alongside rising overweight and obesity prevalence. This shift towards overnutrition is alarming, as it can lead to various non-communicable diseases later in life [72]. Factors associated with overweight, and obesity encompass dietary patterns, including choices such as the consumption of fast food and sweetened drinks, and starchy foods. Additionally, physical inactivity, and various socioeconomic and demographic factors such as age, sex, watching television for more than three hours daily, class, number of children in the family, birth orders play a role in overweight and obesity [24, 33, 58, 73]. A study was conducted to identify individual and contextual factors associated with overweight/obesity among adolescent in two Nigerian state (one northern state one southern state). The finding showed that age (OR 0.86; *P* < 0.001; 95% CI [1.26, 8.70]), physical activity (OR 0.55; *P* = 0.001; 95% CI[0.39, 0.78]) and the upper wealth index (OR 0.47; *P* = 0.018;95% CI [0·25, 0.88]) were inversely related with overweight/obesity, while residing in Southern Nigeria (OR 3.32; *P* = 0·015; 95% CI [1.26, 1.70]), female gender (OR 1·73; *P* = 0·015; 95% CI [1·1, 2·69]) and screen time greater than 2 h per day (O 2·33; *P* = 0·005; 95% CI [1·29, 4·19]) were positively associated with overweight/obesty [13]. Overweight and obesity is one of the key risk factors for many noncommunicable diseases (NCD) such as coronary heart disease, hypertension, an stroked, certain types of cancer, type II diabetes, gallbladder disease, dyslipidemia, osteoarthritis and gout, and pulmonary diseases [74].

The results of the systematic review reveal a concerning pattern of inadequate nutrient intake among adolescents in Southern Nigeria, with a notable absence of studies conducted in Northern Nigeria. The data highlight low levels of essential nutrients, including protein, iron, zinc, calcium, riboflavin, thiamine, niacin, vitamin A, vitamin C, and vitamin D. Despite sufficient carbohydrate intake for the majority of participants, both males and females failed to meet the Recommended Nutrient Intake (RNI) for energy and protein. This deficiency in key nutrients has significant implications for health and well-being. The nutrients play crucial roles in various physiological processes, including growth, immune function, cognitive development, and overall health maintenance [1]. Inadequate intake of these nutrients such as iron can lead to anaemia, resulting in fatigue, weakness, impaired cognitive function and development and reduced physical performance [75]. Similarly, inadequate intake of calcium and vitamin D can compromise bone health and increase the risk of conditions such as osteoporosis later in life [76]. Deficiency of vitamin A, C, and B-complex (thiamine, riboflavin and cobalamin) and folate can weaken immune system, and can lead to a variety of health issues, including scurvy, megaloblastic anaemia and reduced metabolic processes, making more susceptible to infections and other illnesses [77]. Addressing these micronutrients deficiencies requires targeted interventions aimed at improving dietary diversity and nutritional education. Promoting the consumption of nutrient-rich foods such as fruits, vegetables, lean proteins, whole grains, and dairy products can help ensure adequate intake of essential nutrients.

The cumulative findings from these studies indicate a widespread and alarming trend of nutrient deficiencies among the studied population, as nearly everyone had an intake below the expected average requirement (EAR).

In this review, dietary patterns among Nigerian adolescents revealed high consumption of starchy foods, coupled with low intake of fruit, vegetable, and essential nutrients such as proteins, vitamins, and minerals. A systematic review by Wrottesley et al., [6] in Africa also observed transitions towards diets that were increasingly high in energy-dense, processed, and micronutrient-poor foods. Moreover, a systematic review on the dietary intake and practice of adolescent girls in low- and middle-income countries, conducted by Keats et al., [5] reported that the daily consumption of nutritious foods was low among girls; on average, 16% consumed dairy, 46% consumed meats, 44% consumed fruits, and 37% consumed vegetables. In contrast, energy-dense and nutrient-poor foods, such as sweet snacks, salty snacks, fast foods, and sugar-sweetened beverages, were consumed four to six times per week, with averages of 63%, 78%, 23%, and 49% of adolescent girls, respectively. 40% of adolescent girls reported skipping breakfast [5] and a high prevalence of anemia [71]. The effects of food intake on adolescent health include low energy consumption, anemia, weight loss, and obesity [66]. The dietary patterns of adolescents in LMICs and Africa [5, 6] often include high consumption of starchy foods and low intake of fruits, vegetables, and animal proteins. Our review highlighted similar dietary trends among Nigerian adolescents.

The coexistence of undernutrition and overnutrition as observed in our review highlights the triple burden of malnutrition among the adolescent’s population in Nigeria. This burden causes implications such as impaired physical growth and development, leading stunted growth, underweight and thinness and anaemia. These conditions not only affect adolescents’ immediate health but can have lasting effects into adulthood including reduced stature, diminished cognitive function, and increased susceptibility to chronic diseases later in life [72]. The prevalence of both undernutrition and overnutrition among Nigerian adolescents reflect a broader global trend observed in LMICs [3–6, 70]. Many LMICs including those in sub-Saharan Africa, face the dual burden of malnutrition, with high rates of stunting, underweight, and micronutrients deficiencies coexisting with rising rates of overweight and obesity, particularly in urban areas [3, 4, 6, 71]. While the prevalence of overweight remains low compared to undernutrition, it seems that issues related to overnutrition are emerging in Nigeria before the country has fully addressed undernutrition. This trend aligns with global data indicating that the double burden of malnutrition is rising in LMICs [78], driven by rapid economic growth, urbanization, and shifts in dietary habits and physical activity levels. Promoting healthy eating habits, ensuring access to nutritious foods, and implementing nutrition education programs in schools and communities are essential steps toward improving adolescents’ nutritional status and overall health outcomes.

Effective intervention strategies are required to address the burden of malnutrition emerging in Nigeria. In other African countries and low- and middle-income countries (LMICs), evidence from intervention studies has identified food fortification, micronutrient supplementation, and behavior change communication as useful strategies to target thinness, stunting, and vitamin A deficiency [3, 4, 6]. Moreover, recommended interventions include improving the consumption of a balanced diet with an emphasis on locally available and iron-rich foods, promoting healthy dietary habits, creating awareness of the intergenerational effects of malnutrition, raising community awareness, targeted supplementation of iron and folic acid, scaling up facility-based nutrition assessment and counseling programs, and advocating for and promoting food fortification. These recommendations align with the WHO guide for implementing effective actions to improve adolescent nutrition [79]. These efforts will be more effective if global coordination, collaboration, and integration can be achieved [4].

There is a need for a wider range of multisectoral initiatives within African countries, including Nigeria. Effective interventions require multi-sectoral collaborations and community engagement to implement sustainable interventions for improving the nutritional status of adolescents. Support from government and policymakers, health sectors, and nutrition education programs is essential to create an enabling environment for nutritional improvements. Multisectoral initiatives can help equitably improve public health in rapidly growing African cities [80]. This holistic approach includes designing programs that respect and incorporate local cultural practices and dietary habits to ensure community acceptance and effectiveness [81] and implementing interventions that promote long-term changes in dietary habits and food systems rather than temporary fixes. Government support is crucial for developing and enforcing policies, allocating resources, and implementing public health campaigns [82]. The health and education sector can contribute by identifying and addressing nutritional deficiencies, making campaigns and raising awareness about the importance of nutrition and healthy eating habits. By combining efforts from various sectors and engaging the community, sustainable and effective solutions to adolescent nutritional challenges can be achieved.

### Strength and limitation of the review

This systematic review exhibits strength through its comprehensive inclusion of 51 studies, adhering to PRISMA guidelines, conducting a detailed analysis, and employing the NIH checklist for quality assessment. This review significantly contributes to advancing the understanding of the nutritional landscape among Nigerian adolescents.

Despite offering valuable insights, this review is constrained by certain limitations. The majority of the included studies are from Southern Nigeria, potentially limiting the generalizability of findings to the entire country. The scarcity of research in northern regions highlights a gap in understanding nutritional challenges in those areas. Furthermore, there is a potential for publication bias, as the review relies solely on published studies from databases such as PubMed, Scopus and Web of Science. The literature search does not extend to all existing databases, possibly omitting relevant data from other sources, including unpublished studies.

## Conclusion

These findings portray a complex picture of the nutritional challenges faced by this demographic group, highlighting both undernutrition and overnutrition, poor eating behaviour and micronutrient deficiency as significant concerns. There is a high prevalence of underweight, followed by stunting, thinness and overweight while obesity had the least prevalence. The review also identified an inadequate micronutrient intake including iron, zinc, calcium, vitamin A, C, D, niacin, thiamin, riboflavin, cobalamin, and folate. The dietary patterns were characterized by a high consumption of cereals grains and starchy foods, low animal proteins, fast-food with soft drinks, and limited consumption of fruits and vegetables along with meal skipping. The review revealed regional disparities in research representation, with a concentration of studies in Southern Nigeria. This highlights the importance of directing research efforts toward the northern regions, where the prevalence of nutritional issues is equally severe, but less studied.

This systematic review emphasizes the urgent need to address the complex nutritional challenges faced by Nigerian adolescents. Regional disparities, coexistence of undernutrition, and overnutrition require targeted interventions. Improving dietary diversity and nutrition education are essential steps towards ensuring healthy growth and development of this demographic group. Collaborative efforts among the government, healthcare professionals, educators, and communities are necessary to implement effective and sustainable solutions.

### Recommendations

Variations in nutritional outcomes across regions call for region-specific interventions tailored to challenges faced by adolescents in different areas. Promoting diverse dietary patterns rich in essential nutrients, such as proteins, vitamins, and minerals, should be a key focus of nutrition programs. Given the influence of school settings on dietary habits, school-based interventions should be prioritized to promote healthier eating behaviors among adolescents. Educational programs that enhance nutritional knowledge and awareness should be integrated into schools and communities. This integration aims to empower adolescents, with a particular focus on females, enabling them to make informed dietary choices.

### Electronic supplementary material

Below is the link to the electronic supplementary material.

Additional file 1: The data extracted from the 51 studies including authors, date of publication, state where the study conducted, target population, study setting, sample size and key findings.

Additional file 2: Provide NIH Quality Assessment 14 criteria for the study. It shows the score for each study and the total quality.

## Data Availability

No datasets were generated or analysed during the current study.

## Abbreviations

PROSPERO: International Prospective Register of Systematic Reviews
PICO: Population, Intervention, Context, and Outcome
PRISMA: Preferred Reporting Item for Systematic review and Meta-analysis
NIH: National Institutes of Health; RNI: Recommended Nutrient Intake
VAD: Vitamin A Deficiency
EAR: Expected Average Requirement
RNI: Recommended Nutrient Intake
NCD: Non-Communicable Disease
